# Supplementary material for: A Likelihood Approach for Real-Time Calibration of Stochastic Compartmental Epidemic Models
Source: PLoS Comput Biol. 2017 Jan 17;13(1):e1005257. doi: 10.1371/journal.pcbi.1005257 (PMC5240920; doi:10.1371/journal.pcbi.1005257)
Supplement: S1 File — (TAR.GZ) [file pcbi.1005257.s014.tar.gz › HSPH_Online-SI-Revision/Additions/EnKF08/n100-extreme-SEITR_noisy/n100-extreme-SEITR_noisy_EnKF_table0.pdf]

1	R0	{1.38654, 9.85565}	{2.381, 11.5674}	{{1.60106, 3.63066}, {3., 16.5739}}	{71.7225, 48.5374}	inf	5261.	5338.96	{634.221, 12 848.5}	82.3468	0.877572	newly	12 047.4	3245.9	{438., 6868.}	81.1435	0.909729	Reff	0.312221	0.708329	{0.107354, 1.60203}	152.582	ar	92 379.	192 099.	{167 797., 257 973.}	107.947
2	R0	{2.36629, 12.2029}	{2.50763, 6.39422}	{{0.5291, 2.21409}, {3., 10.833}}	{24.5569, 47.6008}	inf	4672.	11 153.5	{5130.33, 17 109.4}	78.9566	0.660594	newly	17 453.	12 479.5	{3931., 23 775.}	69.7319	0.401676	Reff	0.202507	0.776457	{0.306177, 1.02216}	291.708	ar	99 022.	219 078.	{196 860., 271 417.}	121.242
3	R0	{2.32253, 12.836}	{2.38996, 4.82671}	{{1.61136, 3.88522}, {3., 6.40083}}	{28.4329, 62.3971}	inf	6387.	1940.89	{383.8, 3522.57}	94.6529	1.45653	newly	18 266.	2041.67	{265., 3572.}	89.1014	1.18424	Reff	0.29092	0.340799	{0.019391, 0.44663}	62.3299	ar	98 997.	178 861.	{163 640., 234 684.}	81.3955
4	R0	{1.79075, 11.2058}	{2.21935, 6.89737}	{{0.0248204, 2.17219}, {3., 10.5454}}	{61.9883, 38.4483}	inf	6785.	6749.28	{3353.79, 6264.38}	81.5927	0.75473	newly	16 022.	7052.37	{2394., 13 570.}	74.7769	0.580042	Reff	0.341174	0.575342	{0.0200802, 1.05596}	121.333	ar	96 920.	197 932.	{182 927., 209 376.}	104.222
5	R0	{2.26952, 9.62371}	{2.47832, 4.77757}	{{1.58708, 3.607}, {3., 6.1609}}	{28.9652, 50.6}	inf	2810.	2620.9	{301.773, 4930.07}	92.3162	1.36154	newly	16 920.	3430.95	{246., 6135.}	91.9521	1.15018	Reff	0.133902	0.387953	{0.15703, 0.600083}	192.98	ar	98 881.	166 129.	{105 145., 195 634.}	73.8653
6	R0	{1.57191, 6.89004}	{2.14199, 5.60166}	{{0.64077, 2.9748}, {3., 8.34318}}	{74.9015, 37.1046}	inf	4582.	1772.38	{234.095, 2274.07}	92.9833	1.45659	newly	17 405.	1973.96	{127., 5293.}	91.7334	1.42621	Reff	0.240125	0.384188	{0.0596998, 0.491541}	108.979	ar	95 046.	177 276.	{168 427., 191 577.}	86.5162
7	R0	{1.99226, 17.2339}	{2.67578, 8.21394}	{{1.78381, 4.20988}, {3., 13.6591}}	{37.5662, 52.3385}	inf	5638.	3568.63	{739.561, 2918.46}	93.0212	1.28099	newly	11 332.	4098.38	{438., 3925.}	102.249	1.02827	Reff	0.270071	0.547497	{0.111608, 0.978698}	141.419	ar	97 992.	169 354.	{154 701., 193 700.}	72.8244
8	R0	{2.63369, 15.1879}	{2.49698, 6.34151}	{{0.461877, 2.33385}, {3., 9.93388}}	{20.4901, 58.2463}	inf	3741.	6981.83	{2910.27, 8534.87}	83.074	0.892088	newly	14 766.	7742.9	{2076., 11 772.}	75.2828	0.525917	Reff	0.159786	0.838799	{0.397115, 1.73041}	432.273	ar	99 451.	203 477.	{181 254., 273 890.}	104.6
9	R0	{2.53271, 9.90552}	{2.419, 5.05678}	{{1.61794, 3.78488}, {3., 6.34538}}	{22.5226, 48.9499}	inf	2374.	2348.65	{451.335, 5181.18}	92.5109	1.37409	newly	17 411.	3212.38	{352., 6638.}	88.0098	1.14111	Reff	0.11382	0.483753	{0.167698, 0.748076}	327.913	ar	99 355.	192 341.	{176 995., 241 790.}	93.5896
10	R0	{2.48791, 18.9003}	{2.50502, 5.59883}	{{1.46263, 3.60585}, {3., 10.8837}}	{23.4539, 70.377}	inf	6612.	9820.16	{1755.02, 23 810.6}	86.6923	0.972514	newly	13 614.	10 717.1	{1351., 39 497.}	98.7926	0.616903	Reff	0.296509	0.597799	{0.177177, 0.905227}	117.869	ar	99 291.	206 672.	{181 290., 314 415.}	108.148
11	R0	{2.84414, 18.4765}	{2.01794, 8.01164}	{{0.377898, 1.59198}, {3., 18.1725}}	{35.0773, 56.6388}	inf	5335.	7056.6	{1028.77, 9281.87}	87.2531	1.10604	newly	14 675.	7451.15	{787., 12 647.}	93.5315	0.792933	Reff	0.221814	0.368022	{0.0346642, 0.714578}	104.813	ar	99 654.	176 948.	{155 774., 227 794.}	77.5621
12	R0	{2.29369, 14.622}	{2.4675, 12.8166}	{{1.4534, 3.87899}, {3., 18.7139}}	{26.5154, 39.9007}	inf	4308.	9609.	{1991.07, 13 883.3}	81.3917	0.786421	newly	14 159.	7195.87	{1094., 7501.}	92.4928	0.722308	Reff	0.188656	0.7172	{0.305492, 1.00672}	282.331	ar	98 982.	208 106.	{184 892., 253 907.}	110.246
13	R0	{2.73413, 16.7529}	{2.54026, 9.85314}	{{1.92267, 4.11582}, {3., 14.2079}}	{17.3985, 41.1854}	inf	4068.	7014.15	{2545.16, 11 300.8}	82.0412	0.841517	newly	14 704.	7660.72	{1831., 15 460.}	82.5046	0.602727	Reff	0.173699	0.971421	{0.587837, 1.92265}	459.255	ar	99 543.	216 453.	{191 171., 293 995.}	117.446
14	R0	{1.79554, 16.5943}	{2.65134, 11.4521}	{{2.10363, 3.6425}, {3., 17.0472}}	{47.6628, 33.0309}	inf	7555.	2290.56	{294.538, 3708.91}	91.8887	1.26996	newly	11 642.	1595.94	{289., 3213.}	86.2915	1.05281	Reff	0.390135	0.954835	{0.441485, 2.11536}	158.364	ar	96 821.	186 262.	{162 728., 247 990.}	92.3782
15	R0	{1.91429, 13.7771}	{2.71096, 9.24674}	{{2.17417, 4.00387}, {3., 13.7675}}	{41.617, 32.9155}	inf	5519.	4449.2	{1543.33, 5963.75}	84.9968	0.993907	newly	13 230.	3909.97	{858., 11 363.}	85.828	0.861591	Reff	0.264038	0.54598	{0.0524362, 0.997715}	141.705	ar	97 541.	174 404.	{160 049., 216 423.}	78.8011
16	R0	{1.42094, 10.5871}	{2.71597, 4.58459}	{{2.25308, 0.358706}, {3., 6.55912}}	{91.1389, 56.6964}	inf	7009.	6085.46	{1166.36, 14 495.3}	75.8023	0.74355	newly	12 953.	8852.39	{1304., 22 431.}	79.3857	0.564589	Reff	0.417131	0.647363	{0.171976, 1.35179}	87.4752	ar	93 044.	187 480.	{167 542., 230 874.}	101.496
17	R0	{1.45515, 6.16466}	{2.45119, 5.05457}	{{1.59877, 3.90116}, {3., 6.46734}}	{70.3917, 22.177}	inf	6217.	2372.83	{495.743, 2758.99}	89.1644	1.20009	newly	20 574.	2682.25	{388., 2855.}	90.485	1.24069	Reff	0.356002	0.337843	{0.0343812, 0.709066}	59.4189	ar	93 254.	178 669.	{167 973., 199 766.}	92.8729
18	R0	{2.85669, 13.2416}	{2.28636, 5.98344}	{{0.790071, 3.14467}, {3., 8.41068}}	{25.9847, 56.4298}	inf	3137.	2135.76	{494.809, 1624.84}	94.8947	1.55311	newly	17 189.	1684.67	{450., 1312.}	92.4183	1.28871	Reff	0.132179	0.328229	{0.100583, 0.571991}	161.751	ar	99 644.	171 579.	{170 095., 191 245.}	77.2223
19	R0	{2.56417, 17.9558}	{2.74234, 4.1001}	{{2.25576, 0.583646}, {3., 6.30005}}	{13.7879, 77.1656}	inf	6398.	7249.23	{3130.97, 10 225.7}	83.7384	1.00004	newly	14 542.	10 294.	{2153., 13 649.}	90.7633	0.617602	Reff	0.282084	0.520579	{0.111479, 0.843255}	107.784	ar	99 389.	200 093.	{178 422., 250 599.}	101.323
20	R0	{2.27517, 11.1864}	{2.19206, 5.46429}	{{0.711291, 3.26852}, {3., 8.41496}}	{42.8342, 51.1524}	inf	3799.	2535.28	{57.8901, 1661.78}	94.5814	1.75286	newly	16 864.	2068.71	{55., 1583.}	93.4098	1.51297	Reff	0.172117	0.407802	{0.0864587, 0.54026}	158.014	ar	98 894.	177 211.	{168 856., 188 325.}	79.1926
21	R0	{2.35735, 10.4735}	{2.34155, 4.8264}	{{1.61844, 3.82851}, {3., 6.25336}}	{27.9658, 53.918}	inf	3960.	1782.21	{240.218, 2485.67}	94.661	1.5015	newly	18 543.	2009.58	{224., 1990.}	90.2738	1.27488	Reff	0.172039	0.347706	{0.0360973, 0.666063}	130.25	ar	99 036.	178 417.	{167 731., 198 860.}	83.4724
22	R0	{1.79551, 11.9179}	{2.45517, 12.6293}	{{1.47107, 3.98594}, {3., 19.6416}}	{47.4358, 48.5756}	inf	5574.	14 532.1	{1069.22, 58 880.1}	82.8398	0.702978	newly	14 003.	7799.23	{957., 25 052.}	86.909	0.704676	Reff	0.270045	0.569677	{0.106091, 1.05769}	139.581	ar	97 033.	214 142.	{181 421., 316 381.}	120.69
23	R0	{1.23545, 6.31687}	{2.51285, 10.1587}	{{1.9502, 4.08811}, {3., 15.3337}}	{103.395, 80.2557}	inf	4400.	8491.38	{1629.46, 20 384.1}	71.6235	0.505029	newly	13 260.	8623.53	{1199., 21 863.}	93.2446	0.652126	Reff	0.306775	0.899868	{0.510483, 1.95018}	193.332	ar	88 739.	212 351.	{184 868., 300 975.}	139.299
24	R0	{2.24089, 10.1789}	{1.96545, 5.91372}	{{0.69146, 3.14773}, {3., 8.50906}}	{45.3851, 41.9022}	inf	5491.	2555.	{268.103, 2042.63}	94.9123	1.54972	newly	20 012.	2304.38	{214., 2014.}	93.8209	1.39752	Reff	0.242442	0.354202	{0.0804, 0.531898}	95.4383	ar	98 868.	177 670.	{167 175., 191 368.}	79.7047
25	R0	{1.57298, 10.2607}	{2.1135, 5.92378}	{{0.361458, 1.4708}, {3., 18.0584}}	{81.8336, 78.6908}	inf	7361.	6635.56	{1200.82, 6973.36}	93.1775	0.980093	newly	15 457.	6431.87	{499., 9703.}	89.4571	0.823937	Reff	0.403627	0.335049	{0.0628081, 0.693693}	61.2648	ar	94 957.	182 984.	{166 094., 234 771.}	92.7015
26	R0	{1.2154, 8.2576}	{2.52806, 5.81701}	{{1.67518, 3.20404}, {3., 8.14304}}	{108.003, 29.5557}	inf	5404.	2526.96	{790.726, 2429.79}	83.8845	0.97043	newly	11 730.	2779.	{503., 2942.}	83.6593	0.934164	Reff	0.35796	0.431215	{0.208318, 0.724721}	56.2898	ar	88 421.	184 022.	{172 231., 205 638.}	108.121
27	R0	{2.22841, 18.7983}	{2.50582, 5.87313}	{{1.70635, 2.34602}, {3., 7.87519}}	{25.0742, 68.7571}	inf	5503.	6485.45	{684.142, 17 214.}	86.9544	1.12535	newly	11 908.	8597.75	{460., 19 882.}	106.491	0.833927	Reff	0.242317	0.443917	{0.0308747, 1.05874}	130.313	ar	98 756.	188 272.	{168 863., 246 629.}	90.6432
28	R0	{1.27778, 5.36767}	{2.37547, 5.42993}	{{1.59451, 3.86553}, {3., 6.50447}}	{85.9059, 23.2087}	inf	5294.	2075.2	{177.319, 2285.97}	90.7031	1.25527	newly	18 063.	2605.76	{147., 1699.}	94.8649	1.36469	Reff	0.340107	0.35573	{0.00607351, 0.463846}	67.3931	ar	89 843.	179 404.	{169 631., 199 074.}	101.547
29	R0	{2.38042, 15.8901}	{2.37678, 11.4271}	{{1.9523, 4.04567}, {3., 15.6828}}	{26.8348, 28.0864}	inf	6676.	7020.07	{2050.2, 16 404.4}	82.9354	0.907354	newly	15 388.	6299.86	{1497., 9045.}	88.1458	0.753916	Reff	0.307407	0.886994	{0.223907, 1.95228}	205.041	ar	99 112.	202 498.	{179 484., 273 740.}	104.313
30	R0	{2.53188, 12.7607}	{2.10244, 4.47015}	{{0.0886964, 0.067025}, {3., 6.49814}}	{35.8201, 64.9694}	inf	7079.	4216.73	{327.173, 8854.4}	89.2491	1.42479	newly	19 690.	5449.71	{224., 11 124.}	90.4078	1.17988	Reff	0.319143	0.38491	{0.0110045, 0.897506}	76.6363	ar	99 317.	183 248.	{165 869., 234 788.}	86.4311
31	R0	{1.24482, 10.5471}	{2.59315, 11.8143}	{{1.91741, 3.18894}, {3., 18.163}}	{109.32, 56.4867}	inf	5495.	5144.12	{454.394, 9011.07}	84.7465	0.881914	newly	9931.	3451.66	{348., 8708.}	83.166	0.843232	Reff	0.364384	0.957441	{0.346337, 1.92569}	175.709	ar	89 097.	200 551.	{169 258., 255 572.}	125.093
32	R0	{2.30446, 17.9607}	{2.53268, 9.36824}	{{1.51668, 3.65714}, {3., 19.8146}}	{29.0067, 52.3556}	inf	6256.	11 835.4	{1088.89, 28 906.9}	87.9258	0.949213	newly	13 001.	9359.65	{883., 39 647.}	95.5952	0.702842	Reff	0.270705	0.734291	{0.182795, 1.52086}	184.829	ar	99 028.	207 166.	{173 301., 292 611.}	109.199
33	R0	{2.31579, 11.8236}	{2.32265, 5.81436}	{{1.59711, 2.44184}, {3., 10.2378}}	{28.0274, 51.8442}	inf	3005.	8598.83	{521.315, 17 976.7}	79.9863	0.881939	newly	15 166.	10 666.5	{697., 22 559.}	99.6043	0.702068	Reff	0.14124	0.483447	{0.0724952, 1.15043}	257.661	ar	99 012.	192 663.	{171 585., 261 583.}	96.9088
34	R0	{1.6678, 10.6744}	{2.46159, 8.16973}	{{1.49674, 3.66612}, {3., 19.8343}}	{53.4332, 47.6902}	inf	4965.	11 298.	{1520.06, 17 117.5}	81.6513	0.680197	newly	13 645.	12 634.7	{1113., 39 904.}	101.967	0.612196	Reff	0.259643	0.72979	{0.127718, 1.48986}	197.295	ar	95 809.	205 807.	{173 483., 286 353.}	114.81
35	R0	{1.65476, 7.07559}	{2.42105, 5.08377}	{{1.59152, 3.83625}, {3., 6.3117}}	{49.0315, 28.1506}	inf	5229.	3182.96	{195.331, 5500.44}	90.8284	1.34606	newly	19 217.	3357.3	{180., 4632.}	90.1915	1.29808	Reff	0.270404	0.363168	{0.0610218, 0.645647}	71.7011	ar	95 746.	188 284.	{171 629., 212 489.}	102.696
36	R0	{2.71807, 18.5959}	{2.46873, 5.74323}	{{1.52236, 3.58224}, {3., 11.0051}}	{21.5998, 69.1156}	inf	7257.	8947.02	{2013.51, 10 179.7}	86.0355	0.958844	newly	15 024.	10 960.2	{1420., 20 234.}	86.1654	0.558296	Reff	0.322961	0.749623	{0.379661, 0.934718}	134.927	ar	99 540.	198 812.	{175 291., 248 374.}	99.7304
37	R0	{2.6202, 12.7552}	{2.3817, 10.1963}	{{0.381818, 3.87353}, {3., 15.0239}}	{23.8875, 35.9493}	inf	5145.	7639.78	{2283.62, 9445.93}	82.4213	0.935804	newly	18 951.	8507.69	{1657., 10 022.}	96.3697	0.858246	Reff	0.212734	0.746274	{0.128792, 1.46735}	260.96	ar	99 426.	228 444.	{207 424., 284 669.}	129.762
38	R0	{1.47672, 9.81651}	{2.7474, 4.49064}	{{2.23827, 0.198613}, {3., 6.1039}}	{86.0472, 54.2542}	inf	5442.	4021.15	{372.169, 10 372.1}	81.3199	0.928087	newly	13 535.	5241.67	{876., 12 052.}	76.9162	0.711061	Reff	0.299449	0.609451	{0.380668, 0.844382}	108.831	ar	93 815.	171 422.	{157 517., 194 220.}	82.7233
39	R0	{1.90004, 12.3313}	{2.56415, 5.68888}	{{1.48097, 3.59455}, {3., 10.8859}}	{45.1256, 55.0954}	inf	6237.	8431.52	{1428.96, 15 031.5}	81.97	0.824123	newly	15 042.	10 731.	{895., 32 776.}	85.8797	0.598542	Reff	0.303056	0.683506	{0.119897, 1.27229}	145.045	ar	97 413.	202 396.	{175 995., 278 032.}	107.771
40	R0	{2.64218, 18.5237}	{2.56317, 10.6652}	{{1.91825, 4.09915}, {3., 14.6947}}	{17.935, 42.4238}	inf	4328.	5209.8	{1113.14, 7920.88}	86.2411	1.04144	newly	13 194.	5596.64	{867., 8596.}	94.24	0.823447	Reff	0.17626	0.800036	{0.434752, 1.91823}	353.896	ar	99 474.	190 425.	{170 373., 252 772.}	91.4322
41	R0	{1.6945, 7.17996}	{2.30505, 4.79399}	{{1.60729, 3.38578}, {3., 6.36562}}	{55.2521, 33.2309}	inf	7462.	2878.2	{430.273, 2236.52}	93.3446	1.28592	newly	23 349.	3075.91	{319., 2225.}	94.427	1.28282	Reff	0.379415	0.282483	{0.00429892, 0.434175}	48.7345	ar	96 332.	171 613.	{160 834., 206 583.}	79.7109
42	R0	{1.84559, 18.0074}	{2.32671, 9.31524}	{{0.0726534, 3.75354}, {3., 13.5001}}	{64.5021, 48.2699}	inf	6919.	2753.22	{913.669, 2943.2}	94.0635	1.2407	newly	10 541.	1687.35	{466., 1999.}	89.1666	1.01322	Reff	0.338389	0.280307	{0.0190989, 0.465844}	59.4668	ar	97 358.	169 999.	{163 641., 170 514.}	74.6119
43	R0	{2.24965, 17.0361}	{2.60299, 5.83298}	{{1.54049, 3.59572}, {3., 11.0274}}	{28.9328, 65.7611}	inf	7490.	8197.21	{296.275, 13 989.}	90.8101	1.18174	newly	14 237.	9606.5	{304., 30 545.}	104.721	0.854575	Reff	0.339337	0.698363	{0.152841, 0.931935}	119.069	ar	98 770.	189 962.	{167 098., 274 786.}	92.3278
44	R0	{2.54324, 15.7967}	{2.49476, 13.6658}	{{1.48805, 4.223}, {3., 19.6547}}	{23.2653, 37.1086}	inf	3895.	9729.98	{1972.73, 16 609.9}	82.7609	0.843075	newly	14 170.	6032.49	{1046., 6817.}	92.6111	0.787989	Reff	0.167193	0.527571	{0.103491, 0.933965}	230.274	ar	99 354.	191 712.	{169 913., 260 717.}	92.9581
45	R0	{2.54943, 14.8032}	{2.45636, 7.66578}	{{0.743384, 2.47083}, {3., 10.547}}	{22.738, 48.2154}	inf	4134.	7107.53	{3533.99, 8199.83}	81.5506	0.806752	newly	14 996.	7959.32	{2563., 11 115.}	82.2385	0.567258	Reff	0.179352	0.690797	{0.163529, 0.846528}	294.118	ar	99 380.	178 059.	{162 443., 231 270.}	80.7304
46	R0	{2.37755, 12.6461}	{2.39865, 4.99703}	{{1.61437, 3.89343}, {3., 6.58002}}	{26.4538, 60.4856}	inf	5896.	1968.85	{377.706, 2353.07}	94.5697	1.48386	newly	17 991.	2419.92	{286., 1933.}	89.784	1.21935	Reff	0.261673	0.373887	{0.0520311, 0.482404}	77.5607	ar	99 096.	172 295.	{164 345., 196 227.}	79.0071
47	R0	{1.69279, 11.6636}	{2.35807, 5.57692}	{{0.322767, 1.55487}, {3., 17.8199}}	{71.6683, 75.3783}	inf	6849.	7332.09	{647.445, 12 618.3}	93.6955	1.08196	newly	14 547.	8837.21	{713., 13 261.}	104.326	0.837436	Reff	0.3499	0.477676	{0.0672466, 0.81141}	63.7239	ar	96 214.	187 282.	{168 322., 237 660.}	94.6513
48	R0	{1.82402, 10.0038}	{2.55265, 7.27455}	{{0.734326, 2.22082}, {3., 10.9296}}	{52.6955, 35.2138}	inf	6918.	7677.44	{3074.33, 10 780.5}	77.7854	0.716851	newly	17 877.	9448.58	{2330., 24 015.}	82.743	0.607675	Reff	0.343281	0.84627	{0.522628, 2.19572}	153.385	ar	97 076.	198 457.	{174 599., 270 513.}	104.435
49	R0	{1.6424, 11.1667}	{2.46309, 12.1396}	{{1.47335, 3.9181}, {3., 19.7372}}	{55.6543, 54.2383}	inf	4677.	13 582.5	{1398.88, 51 349.9}	84.4909	0.680776	newly	12 499.	9425.76	{1102., 38 784.}	103.875	0.717906	Reff	0.249793	0.623947	{0.261815, 1.46555}	154.963	ar	95 546.	206 368.	{171 302., 327 372.}	115.989
50	R0	{2.05638, 14.3048}	{2.55817, 9.73351}	{{1.93337, 4.11975}, {3., 15.5645}}	{28.9478, 38.3695}	inf	6276.	5644.59	{1864.65, 9270.89}	82.1329	0.87914	newly	14 245.	6850.52	{1369., 8516.}	93.6128	0.738523	Reff	0.290299	0.821543	{0.489269, 1.93335}	184.17	ar	98 194.	210 157.	{190 076., 285 041.}	114.022
